# Editorial: Nutrition to support gut health and the microbiome in athletes

**DOI:** 10.3389/fnut.2023.1207543

**Published:** 2023-05-17

**Authors:** Imran Khan

**Affiliations:** Department of Biotechnology, Faculty of Chemical and Life Sciences, Abdul Wali Khan University Mardan, Mardan, Pakistan

**Keywords:** physical fitness, exercise, gut microbiota, bacteria, prebiotics, probiotics

## Introduction

Symbiotic microorganisms that reside within the gut, commonly referred to as gut microbiota (GM), have been implicated in numerous health and disease-related processes. More recently, there has been growing interest in understanding the potential role of GM in influencing athletic performance. The commensal microorganisms present in the gut, which include archaea, bacteria, and eukaryotes, provide a vast gene pool that is ~1,000 times greater than the number of genes encoded by the human body. These genes support the host by providing a diverse range of metabolic capabilities, nutrient supply, and protection against pathogens (1). Many researchers now believe that a rich and balanced composition of GM is another crucial factor in achieving physical fitness, which was previously thought to be achievable solely through a healthy diet and regular exercise.

Initially, an enriched GM diversity and the presence of specific probiotic bacteria, such as members of the genera *Lactobacilli* and *Bifidobacterium*, were regarded as positive indicators of physical fitness. For example, a group of researchers conducted a comparative analysis of the GM of athletes and non-athletes for diversity indices and discovered that professional rugby players possessed a diverse GM compared to non-athletes. This increased diversity was associated with better overall health and fitness (2).

The scientific understanding of the role of GM in athletic performance has progressed from association studies to targeted studies, which involve manipulating the composition of the GM to enhance athletic performance. Specifically, targeted studies have investigated the use of prebiotics, probiotics, and dietary interventions to modulate GM composition and improve athletic performance. However, research on the GM-athleticism relationship is still in its early stages and limited literature has been documented so far. To gain a better understanding of the mechanisms underlying the GM-athleticism relationship, we launched a Research Topic to provide a unified platform for researchers working on the forefront of this area. After the announcement, we received nine articles, but only four met the standards for publication.

The following section of this manuscript provides a comprehensive summary of the manuscripts that were performed on the role of diet, prebiotics, and probiotics in enhancing physical performance and strength. The upcoming section delves into these aspects in detail, highlighting the current state of knowledge and areas for future research.

## Dietary strategies for enhancing athletic performance

**Figure 1:**
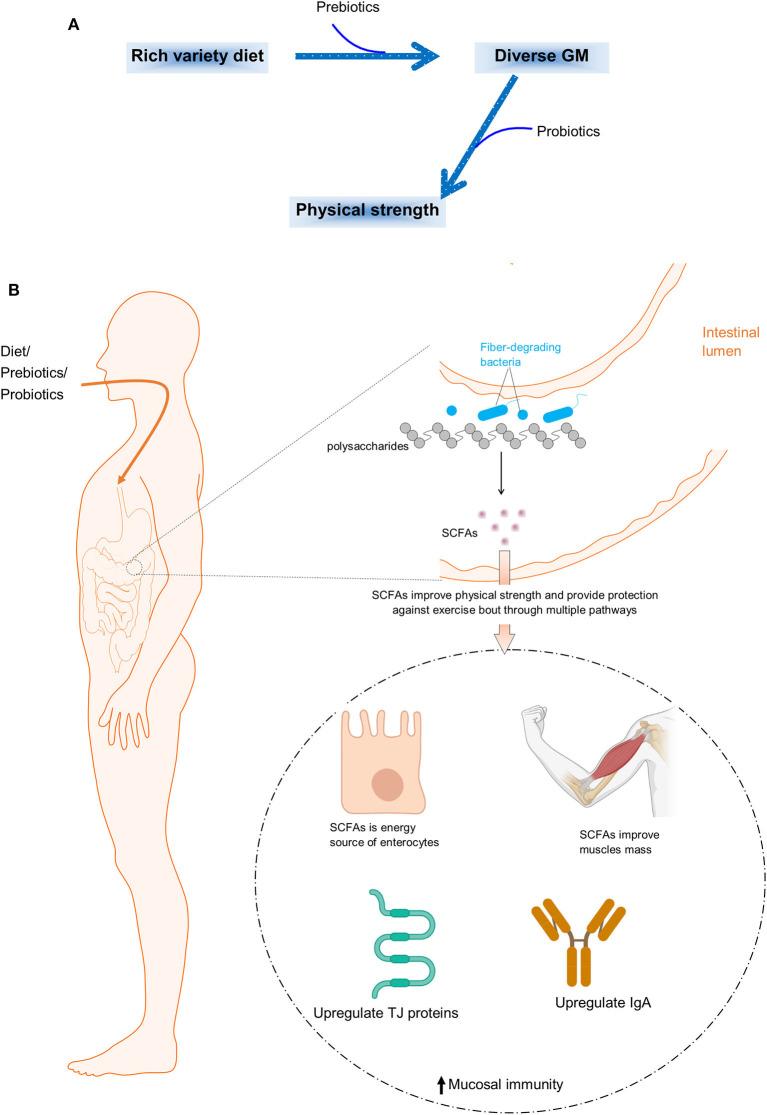
**(A)** Graphical illustration of diet influencing GM diversity and composition, and consequently improving physical exercise. **(B)** Illustration of GM digesting dietary fibers, producing SCFAs as a byproduct, and SCFAs role in improving endurance performance.

Athletes recognize the importance of diet as a critical tool to optimize their fitness and performance. Although significant research has been conducted on nutrition choices for physical wellbeing, there are still unanswered questions. There is mounting scientific evidence that suggests the need to pay closer attention to gut health, particularly if we want to improve our physical strength and performance. Consuming a balanced diet that is comprised of fiber, fruits, and fermented foods can promote the diversity and function of GM, which in turn, can impact exercise performance (Figure 1A).

Diets designed for improving physical strength and performance include the high-protein diet and the carbohydrate-rich diet. The ketogenic diet, which is high in fats and low in carbohydrates is also gaining popularity among athletes due to its potential to enhance fat utilization during exercise, improve endurance, and preserve muscle mass. Although the ketogenic diet has been linked to weight loss and improved insulin sensitivity, its role in enhancing physical performance is not fully understood. To address this knowledge gap, Mancin et al. investigated the effects of a ketogenic Mediterranean diet supplemented with phytoextracts on GM diversity and composition in semi-professional soccer players. Their ketogenic diet, differing from the standard version, had healthy fats, fiber, plant-based protein, and fermented foods as its main components. The authors found that their diet improved exercise performance and supported gut health by promoting the production of short-chain fatty acids (SCFAs) by bacteria from the genera *Odoribacter, Butyricimonas*, and *Ruminococcus*. Other studies have shown that the ketogenic Mediterranean diet can increase the abundance of beneficial bacteria, such as *Akkermansia muciniphila* and *Faecalibacterium prausnitzii*, leading to improvements in gut health, inflammation, and metabolic function (3).

## Achieving physical fitness through prebiotics-induced GM modulation

Prebiotics are dietary fibers that selectively stimulate the growth and activity of beneficial bacteria in the gut. They can increase the abundance of specific beneficial bacteria, such as *Bifidobacterium* and *Lactobacillus*, which produce SCFAs that are important for energy production and immune regulation.

In a recent study, Huang et al. evaluated the prebiotic effect of hyaluronan, a mucopolysaccharide that naturally exists as extracellular matrix in all living organisms (3). After 15 consecutive days of treatment, the mice exhibited enhanced retention time on the accelerating rotarod and carried elevated levels of glycogen and superoxide dismutase in the muscle and liver. Additionally, the level of malondialdehyde in the serum was lower. Hyaluronan was also found to promote the prevalence of xylan/cellulose-degrading bacteria and SCFA-producing bacteria while suppressing the abundance of sulfate-reducing bacteria. By taking *Desulfovibrio vulgaris* as a model for sulfate-reducing bacteria, the authors recorded cytotoxic effects of metabolic products of *Desulfovibrio vulgaris* on the H9c2 cardiomyocytes in a dosage-dependent manner. In addition, metabolic products of *Desulfovibrio vulgaris* also triggered mitochondrial damage by causing mitochondrial fragmentation and depolarization.

Certain types of prebiotics have been shown protective effects against post-exercise infections and intestinal problems. Ruiz-Iglesias et al. found that cocoa fibers could prevent upper respiratory tract infections and gastrointestinal problems after acute intensive exercise. The authors also observed a re-instated level of the lowered IgM contents in the salivary glands and Tγδ CD8αα cells count in Payer's patches. Moreover, cocoa fibers also improved the concentration of SCFAs and SCFAs-producing bacteria which were declined after excessive exercise. In addition, prebiotics can also improve gut barrier function, which may reduce the risk of gastrointestinal distress during exercise (Figure 1B).

## Performance-enhancing probiotics

Supplementation with specific probiotic strains may confer potential benefits, particularly for athletes and fitness enthusiasts. Probiotics, particularly strains belong to the genera *Lactobacillus* and *Bifidobacterium*, have shown promising effects on nutrient absorption and utilization, energy metabolism, and exercise performance. These strains can reduce oxidative stress, inflammation, and fatigue in athletes. Furthermore, probiotic supplementation with these strains has been associated with increased muscle mass and strength in both athletic and elderly populations. In a recent study by Yeh et al. it was demonstrated that *Lactobacillus plantarum* PL-02 supplementation can enhance exercise performance and muscle mass while mitigating exercise-induced increases in lactate and blood ammonia levels in mice. Notably, the combination of PL-02 supplementation and resistance exercise training produced significant benefits in terms of increasing muscle mass and reducing exercise fatigue without inducing physical damage. In another study, it was observed that supplementing with the probiotic *Bacillus subtilis* enhanced performance in female athletes (4). It is also reported that consumption of *Bifidobacterium bifidum* (BIB2) probiotic by sprint athletes significantly improves immune system factors with a positive correlation observed between the duration of consumption and the effect (5).

How do probiotics impact exercise performance? One explanation is that the microorganisms present in the gut produce SCFAs as a byproduct of digesting dietary fiber. SCFAs have been shown to enhance glucose metabolism, boost energy production, and alleviate inflammation, all of which can enhance exercise performance (6). Another possible mechanism is the role of specific bacterial strains, such as *Veillonella atypica*, which have been found to support the host's exercise performance by converting lactate produced during exercise into propionate (7). The precise way in which probiotics impact physical performance is not yet completely understood, but there is a strong link between the two.

## Conclusion

GM may play a critical role in regulating energy metabolism and modulating immune system function, both of which are vital for optimal exercise performance. Recent studies have demonstrated that manipulation of the GM, such as the introduction of probiotics or dietary changes, can positively impact exercise outcomes. Understanding the complex interplay between GM and exercise may significantly benefit athletes. Optimizing athletes' GM through targeted dietary interventions could improve their stamina, lower inflammation, and support physical fitness. As our understanding of this relationship grows, it is likely that we will witness innovative ways to leverage this knowledge to optimize human wellbeing and physical performance.

## Author contributions

This editorial is designed and written by IK.
